# Association between Gene Polymorphisms of Vitamin D Receptor and Gestational Diabetes Mellitus: A Systematic Review and Meta-Analysis

**DOI:** 10.3390/ijerph18010205

**Published:** 2020-12-29

**Authors:** Qian Zhou, Shiwu Wen, Miao Liu, Sulei Zhang, Xin Jin, Aizhong Liu

**Affiliations:** 1 Department of Epidemiology and Health Statistics, Xiangya School of Public Health, Central South University, Changsha 410078, China; zhqian@csu.edu.cn (Q.Z.); liumiao@csu.edu.cn (M.L.); 186912052@csu.edu.cn (S.Z.); 2 Hunan Provincial Key Laboratory of Clinical Epidemiology, Xiangya School of Public Health, Central South University, Changsha 410078, China; 3 OMNI Research Group, Ottawa Hospital Research Institute, Ottawa, ON K1H 8M5, Canada; swwen@ohri.ca; 4 Department of Obstetrics and Gynaecology, School of Epidemiology and Public Health, University of Ottawa Faculty of Medicine, Ottawa, ON K1H 8M5, Canada; 5 Department of Maternal and Child Health, Xiangya School of Public Health, Central South University, Changsha 410078, China; jinx0423@csu.edu.cn

**Keywords:** vitamin D receptor genes, gene polymorphism, gestational diabetes mellitus

## Abstract

(1) Background: Studies on the association between Vitamin D receptor gene polymorphism and gestational diabetes mellitus have been inconsistent. The aim of this study was to summarize available evidence on the association between polymorphisms of Vitamin D receptor genes and susceptibility to gestational diabetes mellitus. (2) Methods: We searched databases of PubMed, Web of Science, Embase, China national knowledge infrastructure (CNKI), China science and technology journal database (VIP), and Wanfang Data for relevant articles. A systematic review and a meta-analysis were done to compare the distribution of Vitamin D receptor gene polymorphisms in gestational diabetes mellitus patients with those in controls using allelic, codominant, dominant, and recessive models. (3) Results: A total of eight eligible articles were included in the systematic review and of them, six articles were included in the meta-analysis. The vitamin D receptor gene rs7975232 polymorphism was associated with gestational diabetes mellitus under the allelic model (odds ratio = 1.28, 95% confidence interval 1.06–1.56), codominant model (CC vs. AA odds ratio = 1.97, 95% confidence interval 1.28–3.05), and recessive model (odds ratio = 1.83, 95% confidence interval 1.27–2.64) in the case of low heterogeneity. High heterogeneity existed in studies on the association of vitamin D receptor genes *rs1544410*, *rs2228570*, and *rs731236* with gestational diabetes mellitus, and the most common sources of heterogeneity were the year of publication and matching. (4) Conclusion: Polymorphism of the vitamin D receptor gene *rs7975232* may be associated with risk of developing gestational diabetes mellitus. Future studies should be designed to include standardized data collection and matching for important confounding factors such as body mass index, age, and race.

## 1. Introduction

Gestational diabetes mellitus (GDM), a pregnancy complication, is defined as any degree of glucose intolerance that first develops or is first diagnosed during pregnancy [1]. The reported rates of GDM in the world range from 9.3% to 25.5% due to differences in diagnostic criteria, screening methods, and other factors [2,3,4,5]. GMD is associated with adverse pregnancy outcomes such as preeclampsia, cesarean section, birth injury, and large size for gestational age of infants [6,7]. Although glucose tolerance usually returns to a normal level once the baby is delivered, women diagnosed with GDM have an increased risk of developing type 2 diabetes, especially in the first 5 years after childbirth [8]. Women affected by GDM have a two-fold higher risk of cardiovascular events after childbirth as compared with women without GDM [9].

The incidence of GDM has been increasing. However, the biomolecular mechanisms of GDM are not clearly understood [10,11]. It is therefore difficult to develop effective prevention strategies based on solid scientific evidence. Recent reviews have suggested that vitamin D deficiency may play a role in the molecular basis of insulin resistance [12,13]. The prevalence of vitamin D deficiency (serum 25(OH)D levels < 50 nmol/L or 20 ng/mL) in pregnant women ranges from 46% to 87% across different countries and regions, which indicates that vitamin D deficiency during pregnancy may be a common health problem worldwide [14]. Vitamin D displays cellular activities after binding to the vitamin D receptor (VDR), a member of the steroid hormone receptor superfamily [15]. The VDR gene is localized on chromosome 12q12–14 [16] and the role of single nucleotide polymorphisms (SNPs) in the VDR gene has been investigated in many diseases [17,18]. VDR has been found to be strongly expressed in pancreatic beta cells [19]. Previous studies have reported that VDR gene polymorphisms are connected with insulin resistance, which could reflect the changing of insulin sensitivity [20], and with insulin secretory capacity [21].

Genetic variants in the VDR gene are potential candidates for investigation to better understand how vitamin D pathways are involved in the pathogenesis of GDM. Results from studies on the relationship between VDR gene polymorphisms and GDM are inconsistent. The aim of this review was to summarize the available evidence on the association between VDR gene polymorphisms and GDM.

## 2. Methods

### 2.1. Search Strategy

This systematic review/meta-analysis was performed according to the Preferred Reporting Items for Systematic Reviews and Meta-Analyses (PRISMA) guidelines [22]. A systematic literature search was done based on the English electronic databases of PubMed, Web of Science, and Embase, and the Chinese electronic databases of CNKI, VIP, Wanfang Data from their inception dates to April 2020. Articles in English and in Chinese were searched and the search strategy was adjusted across databases. Specifically, for the databases of PubMed and Embase, the search terms were: (“Vitamin D receptor gene “OR “VDR gene”) AND (“gestational diabetes mellitus”). For the databases of Web of Science and CNKI, the search terms were: “Vitamin D receptor gene, VDR gene” [Mesh] AND “gestational diabetes mellitus” [Mesh]. For the database of Wanfang Data, the search terms were: “Vitamin D receptor gene” [Mesh]” gestational diabetes mellitus” [Mesh] AND “VDR gene” [Mesh]” gestational diabetes mellitus” [Mesh]; for the database of VIP, the search terms were: (“Vitamin D receptor gene” [Title/Abstract] OR “VDR gene” [Title/Abstract]) AND (“gestational diabetes mellitus” [Title/Abstract]). Search terms were changed for different databases in order to obtain the most comprehensive and accurate search results according to the characteristics of each electronic database.

### 2.2. Eligibility Criteria

Studies were included in this systematic review if they met the following criteria: (1) observational study or experimental study which was conducted in pregnant women; (2) the study investigated the association between VDR gene polymorphism and GDM; (3) diagnoses for GDM were reported; (4) sample size was reported in article; (5) full article was written in English or Chinese. Exclusion criteria were as follows: (1) lack of information on specific SNPs of VDR gene; (2) no available information on distribution of VDR gene polymorphism in case and control; (3) abstracts, case reports, comments, conferences, reviews, academic dissertations, and book chapters; (4) case and control were not pregnant women with GDM and without GDM. When data from the same population were published several times, the first published paper was included.

### 2.3. Data Extraction

Endnote was used to manage articles and two investigators (Q.Z. and X.J.) assessed articles and extracted relevant data from eligible articles independently. Articles that met the inclusion requirements after screening for titles and abstracts were selected for full-text review. The following data were extracted from the eligible original studies: the last name of the first author, year of publication, diagnostic criteria, country of study, study design, the method used to evaluate VDR gene polymorphisms, sample size, age of study participants, matching, the result of Hardy–Weinberg equilibrium (HWE) in the control group, number of participants for each genotype, and the SNP code of the VDR gene that was studied.

### 2.4. Quality Assessment

The NEWCASTLE-OTTAWA QUALITY ASSESSMENT SCALE was used to assess the quality of included studies (Supplementary 1). In addition, we selected body mass index (or BMI, weight in kg/height in m^2^) as the most important factor when assessing the comparability of cases and controls for this meta-analysis, because it has been reported in a number of studies that BMI is significantly associated with GDM [23,24]. Two investigators completed quality assessment independently.

### 2.5. Statistical Analysis

Statistical analyses were performed using the “meta” package of software R (version 3.5.1) (AT&T, Dallas, TX, USA) Summary odds ratios (ORs) with their 95% confidence intervals (Cis) in the allelic genetic model, codominant genetic model, dominant genetic model, and recessive genetic model were calculated to assess the strength of association between VDR gene polymorphisms and the susceptibility to GDM. Cochran’s χ2 test and the *I^2^* statistic were used to evaluate the heterogeneity across studies, with *I^2^* ≥ 25%, *I^2^* ≥ 50%, and *I^2^* ≥ 75%, respectively, indicating low, moderate, and high heterogeneity. The summary ORs were calculated using the Mantel–Haenszel method with a random-effects model if the heterogeneity observed across studies was significant (*p* ≤ 0.10 and/or *I^2^* > 50%). Otherwise, a fixed-effects model was used. HWE was assessed by Pearson’s chi-squared test using the “meta” package of software R version 3.5.1. If the included article did not report the HWE in the control group, it was considered as in HWE if *p* > 0.05.

Because the number of included studies was less than 10, no formal assessment of publication bias was performed. Subgroup analysis and sensitivity analysis were conducted when heterogeneity was moderate or high (*I^2^* ≥ 50%). Subgroup analysis was carried out according to the following features of the included studies: year of publication (before 2019 versus 2019), study design (conventional case–control study versus not conventional case–control study), diagnostic criteria (International Association of Diabetes & Pregnancy Study Groups (IADPSG) versus American Diabetes Association (ADA), and matching (yes versus no).

## 3. Results

### 3.1. Literature Search

**Figure 1:**
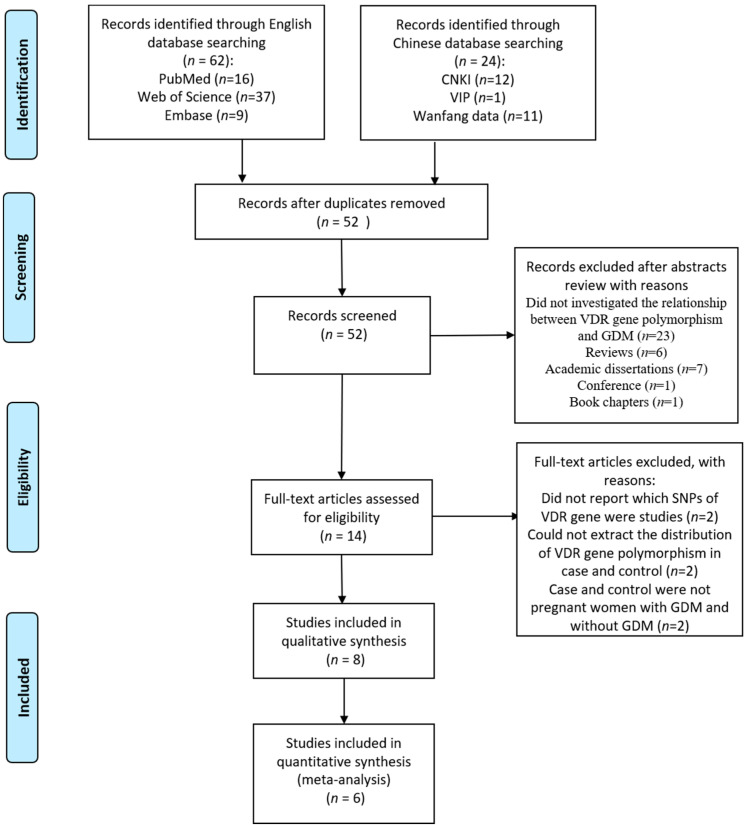
Preferred Reporting Items for Systematic Reviews and Meta-Analyses (PRISMA) flow chart of study identification process.

The initial search identified 84 articles and finally, a total of eight eligible articles were included in the review, with six articles included in the meta-analysis (Figure 1). Among the 14 fully reviewed articles, 2 were excluded for not reporting which SNPs of the VDR gene were studied, 2 were excluded for lacking information on the distribution of VDR gene polymorphism, and another 2 were excluded because the comparison was not between GDM and non-GDM women. SNPs of the VDR gene reported in two articles were not examined in other studies, so these two articles were included in the meta-analysis.

### 3.2. Characteristics of Eligible Articles

Characteristics of the eight included articles are presented in Table 1. All included studies were case–control studies. These studies were conducted in five countries with 1647 cases and 1993 controls. Among the eight eligible studies, four were published in 2019 and the other four were published earlier. The quality assessment scores indicated that articles published in more recent years tended to have higher quality.

### 3.3. Association between VDR Gene Polymorphism and GDM

**Figure 2:**
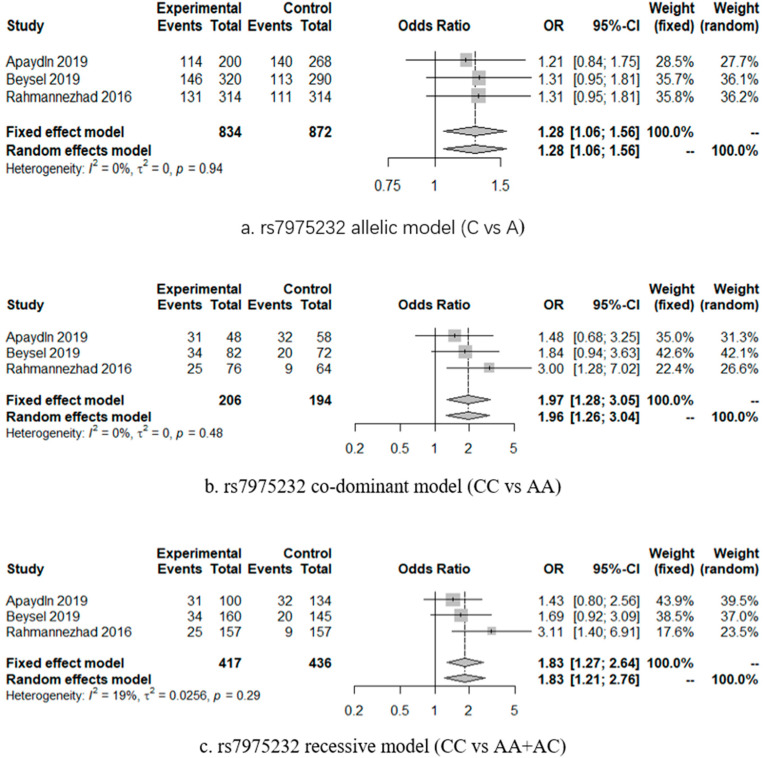
Forest plots of the significant results. (**a**) *rs7975232* allelic model (C vs. A); (**b**) *rs7975232* co-dominant model (CC vs. AA); (**c**) *rs7975232* recessive model (CC vs. AA + AC).

Table 2 displays the summarized association between VDR gene polymorphism and GDM. The results showed that VDR gene *rs7975232* polymorphism was associated with GDM significantly under the allelic model (OR = 1.28, 95% CI = 1.06–1.56), codominant model (CC vs. AA OR = 1.97, 95% CI = 1.28–3.05, P = 0.002), and recessive model (OR = 1.83, 95% CI = 1.27-2.64, *p* = 0.001) in the case of low heterogeneity. Figure 2 displays the forest plot of these results. High heterogeneity existed in studies on the association of VDR genes *rs1544410*, *rs2228570*, and *rs731236* with GDM.

Two eligible studies examined the association of VDR genes *rs739837*, *rs11574143*, and *rs10735810* with GDM. However, these SNPs were not reported in other eligible studies. As a result, these two studies were not included in meta-analysis. These two articles reported no significant difference in VDR genes *rs739837*, *rs11574143*, and *rs10735810* between cases and controls.

### 3.4. Subgroup Analysis and Sensitivity Analysis

The summarized sources of heterogeneity are displayed in Table 3. The results indicated that matching and year of publication were the most common sources of heterogeneity for VDR genes *rs1544410*, *rs2228570* and *rs731236*. Study design and the diagnostic criteria were the main sources of heterogeneity of VDR genes *rs2228570*, *rs731236*. The results of sensitivity analysis are shown as forest maps in Supplementary 2. For *rs1544410*, the results fluctuated greatly after omitting El-Beshbishy 2015 [25] and for *rs731236*, the results fluctuated greatly after omitting Rahmannezhad 2016 [26] or Zhu 2019 [27].

## 4. Discussion

It has been hypothesized that deficiency of vitamin D may be a risk factor for GDM, but findings in previous studies on this topic remain inconsistent [33,34,35,36]. There is a hypothesis that the association observed between vitamin D deficiency and the risk of GDM might be confounded by seasonality which is not easy to control or measure [37,38]. On the other hand, the Ma’anshan birth cohort study suggested that VDR variants rather than vitamin D concentrations were associated with the risk of GDM [26]. Compared with vitamin D concentrations, VDR variants are not affected by seasonality, and could therefore be a better way to understand how vitamin D pathways are involved in the pathogenesis of GDM. The inconsistency of the results from studies on the relationship between VDR gene polymorphisms and GDM may be due to the following reasons: first, the sample size of studies varies greatly, resulting in different research effectiveness; second, the distribution of the vitamin D receptor gene may be different between races, which could introduce bias to evaluation of the association between polymorphisms of vitamin D receptor genes and susceptibility to gestational diabetes mellitus; third, the quality of the studies, the control of confounders, and other factors that contribute to the heterogeneity may make the results of previous studies inconsistent. This systematic review and meta-analysis synthesized the available evidence on the association between VDR gene polymorphisms and GDM. Eight eligible studies conducted in five countries with a total of 1647 cases and 1993 controls were included. Among the eight studies, six were included in the meta-analysis (with 883 cases and 1115 controls). The results of meta-analysis showed that VDR gene *rs7975232* polymorphism was correlated with GDM under several genetic models, while VDR gene *rs1544410*, *rs2228570*, and *rs731236* polymorphisms were uncorrelated with GDM. The results should be interpreted with caution, however, because the sample size was not large enough, and there was high heterogeneity among studies on VDR gene polymorphisms *rs1544410*, *rs2228570*, and *rs731236*. Although six studies were included in the meta-analysis, the number of studies for each SNP was less than that, so the sample size for each specific VDR gene polymorphism was limited. Our subgroup analysis found that year of publication was one of the most common sources of heterogeneity among studies on VDR gene polymorphisms *rs1544410*, *rs2228570*, and *rs731236*. It should be noted that all eligible studies were published after 2012 and three of them were published in 2019, indicating that this field of research may be still in its infancy. Another meta-analysis published recently about the association between GDM and seven gene polymorphisms reported that the VDR *FokI rs2228570* polymorphism was significantly associated with susceptibility to GDM [39]. This is contrary to our findings, maybe due to differences in the inclusion criteria used in that review. The authors included both *rs2228570* and *rs10735810* polymorphisms of the VDR gene in the meta-analysis, which probably have the same restriction enzyme *FokI*.

Currently, there are no effective measures to prevent GDM. However, the relationship between VDR gene polymorphism and gestational diabetes susceptibility may provide new ideas for the prevention of gestational diabetes in the future. Populations susceptible to GDM during pregnancy could be found by genotyping of VDR, so as to provide targeted vitamin D supplementation recommendations. Although no definitive conclusion regarding the association between VDR gene polymorphism and GDM could be drawn from this systematic review and meta-analysis, important information for future research directions could be obtained from this exercise. First, the VDR gene *rs7975232* was the most likely to be associated with GDM, but only three studies have examined the gene polymorphism of *rs7975232*, with a total of only 417 cases and 436 controls. Therefore, future studies should focus on the VDR gene *rs7975232*. Second, although the associations of VDR genes *rs1544410*, *rs2228570*, and *rs731236* with GDM were not significant overall, subgroup analysis indicated that matching and year of publication were the most common sources of heterogeneity, suggesting that the quality of study has been improving in recent years and that future studies should try to control confounding by matching important factors such as BMI, age, and race. Third, important information such as SNPs of the VDR gene [40,41] or distribution of VDR gene polymorphism [42,43] was not reported in some of the eligible studies, which made inclusion of these studies in the meta-analysis difficult. If data collection can be standardized in the future, more studies will be able to contribute to meta-analyses.

## 5. Limitations

Our study had several limitations. First, we searched for publications in English and Chinese only, and would have missed articles in other languages or gray literature. Second, the heterogeneity of the subgroup analysis was high, indicating that interpretation of the results of this study should be treated with caution. Third, race is a very important factor during polymorphism analysis. However, as not all included studies reported the race of the study population, the lack of data made it impossible to conduct subgroup analysis using race as a grouping factor.

## 6. Conclusions

This systematic review suggests that the *rs7975232* polymorphism of the VDR gene may be associated with GDM. Future studies should use standardized data collection and matching for important confounding factors such as BMI, age, and race.

## Figures and Tables

**Table 1:** Characteristics of the eligible studies.

Study	Country	Study Design	Diagnostic Criteria	Assessment of Gene Polymorphism	Match	SNPs	HWE in Control	Quality Assessment Score	Case		Control	
									*n*	Age	*n*	Age
Apaydln, 2019 [25]	Turkey	case–control	IADPSG *	Sanger-based DNA sequencing	not reported	*rs2228570 rs1544410 rs7975232 rs731236*	in HWE	7	100	29.41 ± 5.02	135	29.13 ± 5.20
Zhu, 2019 [26]	China	nested case–control	ADA **	improved multiple ligase detection reaction	age	*rs1544410 rs731236*	not reported	6	274	not reported	380	not reported
Beysel, 2019 [27]	Turkey	case–control	IADPSG *	Real-time PCR	age, gestational age	*rs2228570 rs1544410 rs7975232 rs731236*	in HWE	6	160	29.35 ± 5.36	145	29.35 ± 5.36
El-Beshbishy, 2015 [28]	Saudi Arabia	cross-sectional	ADA **	PCR-RFLP ***	not reported	*rs2228570 rs1544410*	not reported	6	112	41 ± 4.1	218	40 ± 3.1
Rahmannezhad, 2016 [29]	Iran	case–control	IADPSG *	PCR-RFLP ***	not reported	*rs7975232 rs731236*	not reported	5	157	29.11 ± 4.6	157	28.19 ± 4.05
Qi, 2013 [30]	China	case–control	ADA **		not reported	*rs1544410*	in HWE	4	80	28.26 ± 3.15	80	27.52 ± 2.21
Wang, 2015 [31]	China	nested case–control	ADA **	TaqMan allelic discrimination assays	race, gestational age, BMI	*rs739837 rs11574143*	in HWE	5	692	32.00 (30.00, 35.00) ****	802	31.00 (28.00, 34.00) ****
Siqueira, 2019 [32]	Brazil	prospective case–control	IADPSG *	PCR-RFLP ***	not reported	*rs10735810*	in HWE	7	72	33 ± 5.7	76	30 ± 6.7

* International Association of Diabetes & Pregnancy Study Groups; ** American Diabetes Association; *** polymerase chain reaction and restriction fragment length polymorphism; **** Data are shown as medians (interquartile range) for the quantitative variables with non-normal distribution.

**Table 2:** Association of vitamin D receptor (VDR) gene polymorphisms and gestational diabetes mellitus (GDM) characteristics as reported in the eligible studies.

	Participants with GMD (*n*)	Controls (*n*)	OR (95% CI)	*p*	Heterogeneity			Effect Model
					Q Statistic (DF; *p* Value)	τ2	*I^2^*	
*rs7975232*	417	436						
Allelic (C vs. A)			1.28 (1.06,1.56)	0.012 ^a^	0.13 (2, 0.9379)	0	0.0%	fixed
Codominant (AC vs. AA)			1.04 (0.76,1.42)	0.808	0.34 (2, 0.8425)	0	0.0%	fixed
Codominant (CC vs. AA)			1.97 (1.28,3.05)	0.002 ^a^	1.47 (2, 0.4787)	0	0.0%	fixed
Dominant (AC + CC vs. AA)			1.20 (0.89,1.62)	0.232	0.20 (2, 0.9037)	0	0.0%	fixed
Recessive (CC vs. AA + AC)			1.83 (1.27, 2.64)	0.001 ^a^	2.46 (2, 0.2920)	0.026	18.8%	fixed
*rs1544410*	727	957						
Allelic (G vs. A)			0.758 (0.391, 1.469)	0.412	48.59 (4, <0.0001)	0.508	91.8%	random
Codominant (AG vs. AA)			0.762 (0.308, 1.888)	0.557	33.99 (3, <0.0001)	0.782	91.2%	random
Codominant (GG vs. AA)			0.523 (0.109, 2.504)	0.417	28.56 (2, <0.0001)	1.776	93.0%	random
Dominant (AG + GG vs. AA)			0.740 (0.274, 2.002)	0.553	45.75 (3, <0.0001)	0.963	93.4%	random
Recessive (GG vs. AA + AG)			0.624 (0.274, 1.421)	0.261	17.23 (3, 0.0006)	0.577	82.6%	random
*rs2228570*	372	497						
Allelic (T vs. C)			1.333 (0.852, 2.085)	0.209	9.67 (2,0.0079)	0.124	79.3%	random
Codominant (CT vs. CC)			1.070 (0.593, 1.929)	0.583	6.50 (2, 0.0388)	0.188	69.2%	random
Codominant (TT vs. CC)			1.612 (0.672, 3.865)	0.285	8.87 (2, 0.0118)	0.458	77.5%	random
Dominant (CT + TT vs. CC)			1.230 (0.659, 2.293)	0.516	8.67 (2, 0.0131)	0.233	76.9%	random
Recessive (TT vs. CC + CT)			1.579 (0.907, 2.748)	0.030	4.68 (2, 0.0965)	0.135	57.2%	random
*rs731236*	683	793						
Allelic (C vs. T)			1.141 (0.757, 1.720)	0.528	12.97 (3, 0.0047)	0.131	76.9%	random
Codominant (CT vs. TT)			1.000 (0.541, 1.848)	0.999	15.11 (3, 0.0017)	0.314	80.1%	random
Codominant (CC vs. TT)			1.118 (0.753, 1.661)	0.581	2.52 (2, 0.2834)	0.035	20.7%	fixed
Dominant (CT + CC vs. TT)			1.062 (0.599, 1.886)	0.836	15.51 (3, 0.0014)	0.275	80.7%	random
Recessive (CC vs. TT + CT)			1.236 (0.848, 1.802)	0.269	0.80 (2, 0.6708)	0	0.0%	fixed

^a^*p* < 0.05; OR: odds ratio; CI: confidence interval.

**Table 3:** The sources of heterogeneity.

SNPs	Eligible Studies	Allelic	Codominant (Mutant Heterozygote vs. Wild Homozygote)	Codominant (Mutant Homozygote vs. Wild Homozygote)	Dominant	Recessive
*rs1544410*	Apaydln, 2019 Zhu, 2019 Beysel, 2019 El-Beshbishy, 2015 Qi, 2013	year of publication, match or not	year of publication, match or not	year of publication, match or not	year of publication, match or not	year of publication, study design, diagnostic criteria
*rs2228570*	Apaydln, 2019 Beysel, 2019 El-Beshbishy, 2015	year of publication, study design, diagnostic criteria	not found	year of publication, study design, diagnostic criteria	match or not	match or not
*rs731236*	Apaydln, 2019 Zhu, 2019 Beysel, 2019 Rahmannezhad, 2016	year of publication, study design, diagnostic criteria, match or not	year of publication, study design, diagnostic criteria, match or not	__	year of publication, study design, diagnostic criteria, match or not	__

## Data Availability

The statement is not needed.

## Supplementary Materials

The following are available online at https://www.mdpi.com/1660-4601/18/1/205/s1, Supplementary 1: Newcastle—Ottawa quality assessment scale—Case control studies; Supplementary 2: The results of sensitivity analysis.
