# Supplementary material for: Association between Gene Polymorphisms of Vitamin D Receptor and Gestational Diabetes Mellitus: A Systematic Review and Meta-Analysis
Source: Int J Environ Res Public Health. 2020 Dec 29;18(1):205. doi: 10.3390/ijerph18010205 (PMC7794905; doi:10.3390/ijerph18010205)

The results of sensitivity analysis.

rs1544410 allelic model (G vs A)


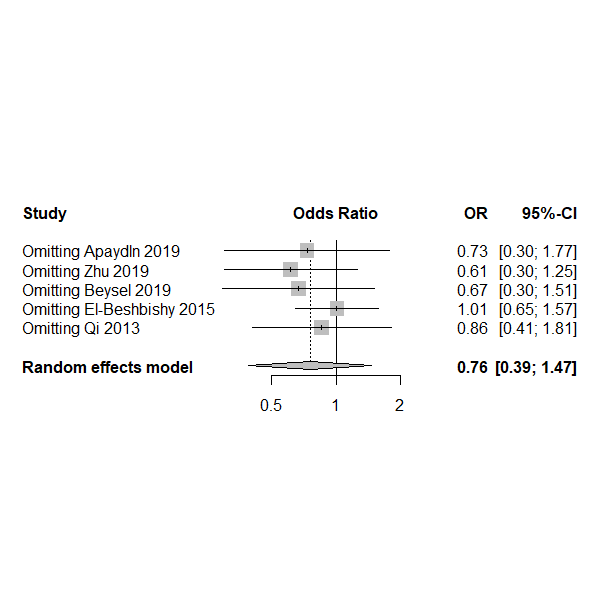


rs1544410 co-dominant model (AG vs AA)


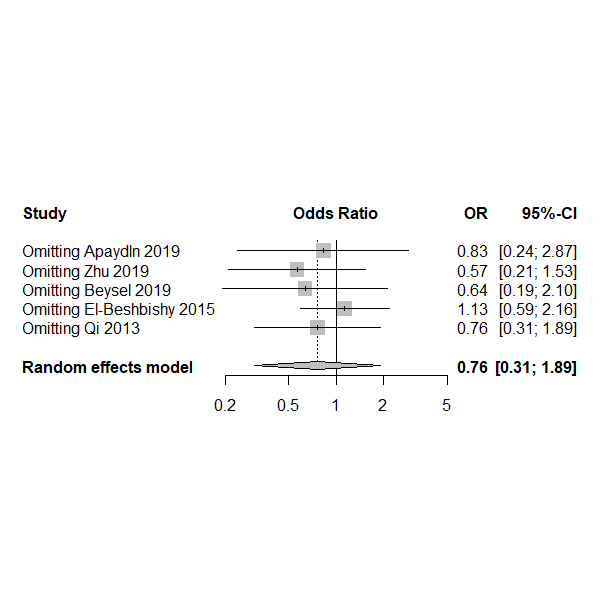


rs1544410 co-dominant model (GG vs AA)


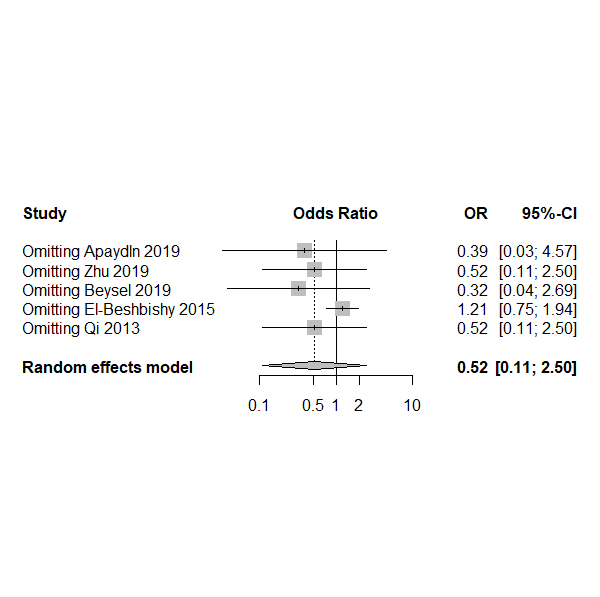


rs1544410 dominant model（AG+ GG vs AA）


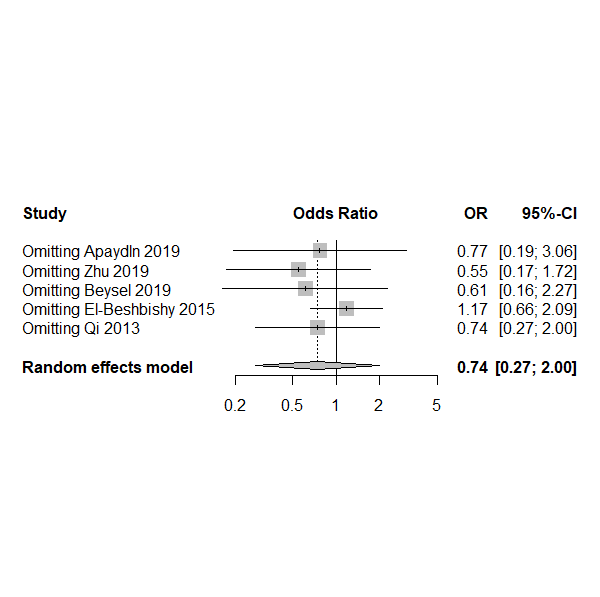


rs1544410 recessive model (GG VS AA+AG)


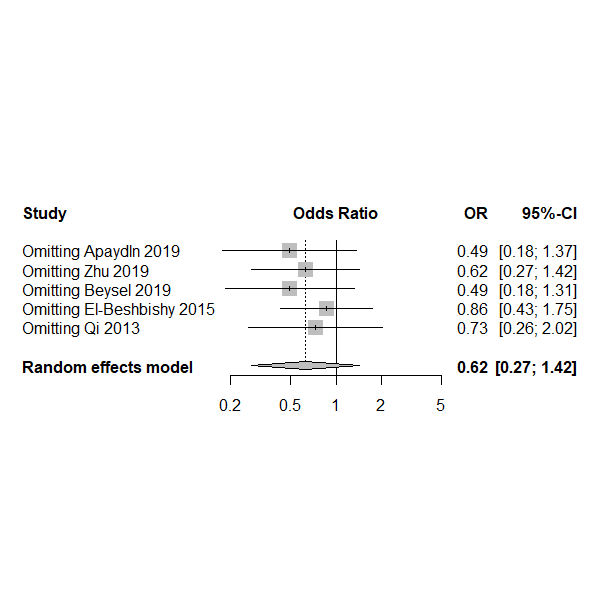


rs2228570 allelic model (T vs C)


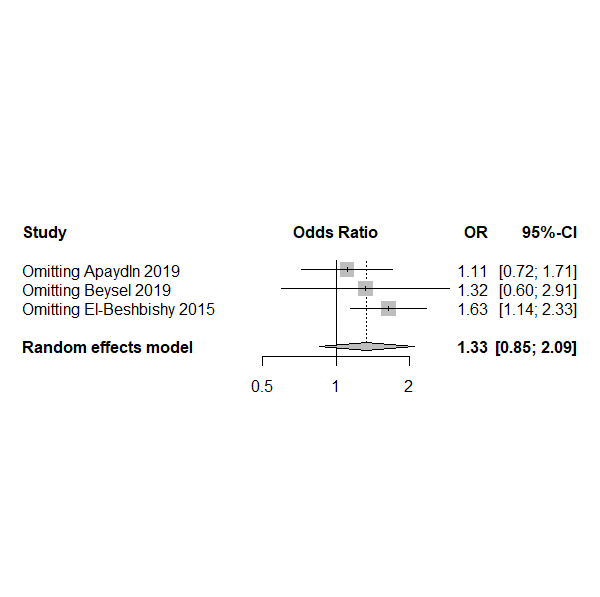


rs2228570 co-dominant model (CT vs CC)


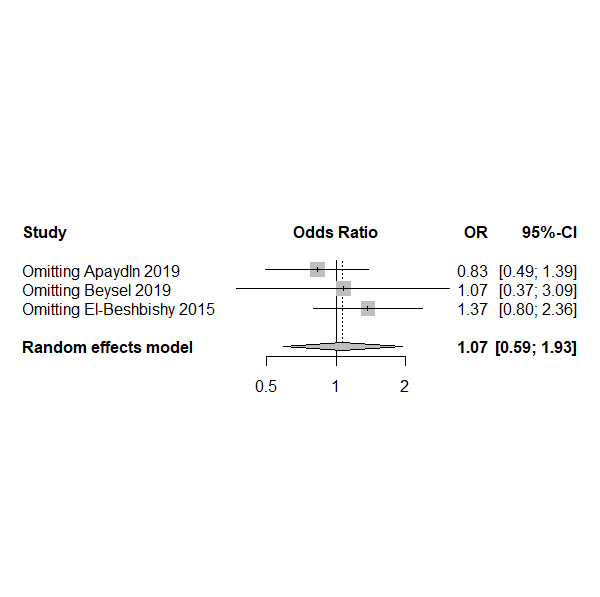


rs2228570 co-dominant model (TT vs CC)


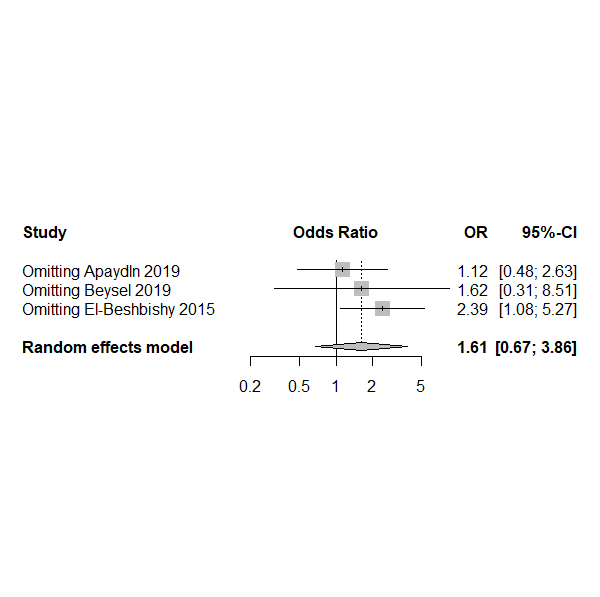


rs2228570 dominant model (TT vs CC+CT)


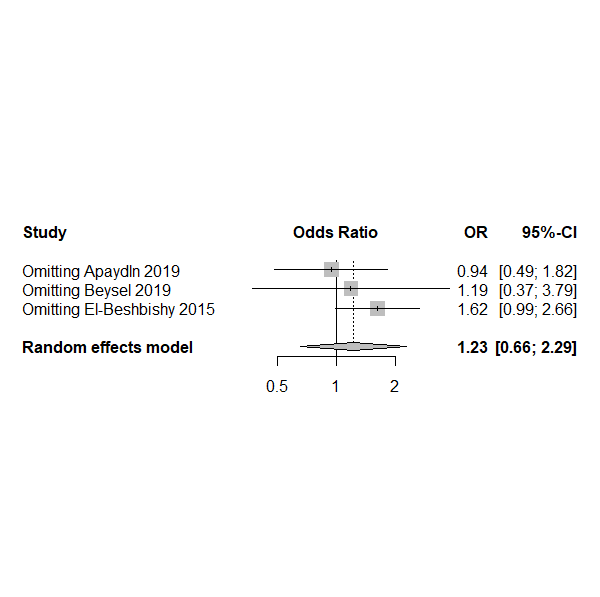


rs2228570 recessive model (TT vs CC+CT)


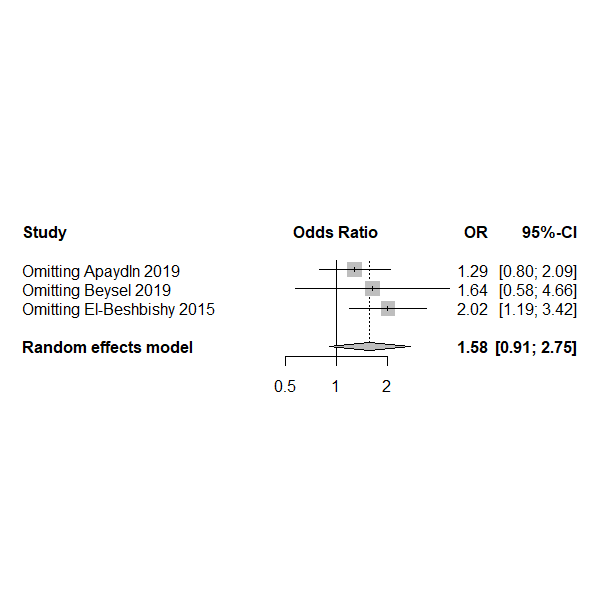


rs731236 allelic model (C vs T)


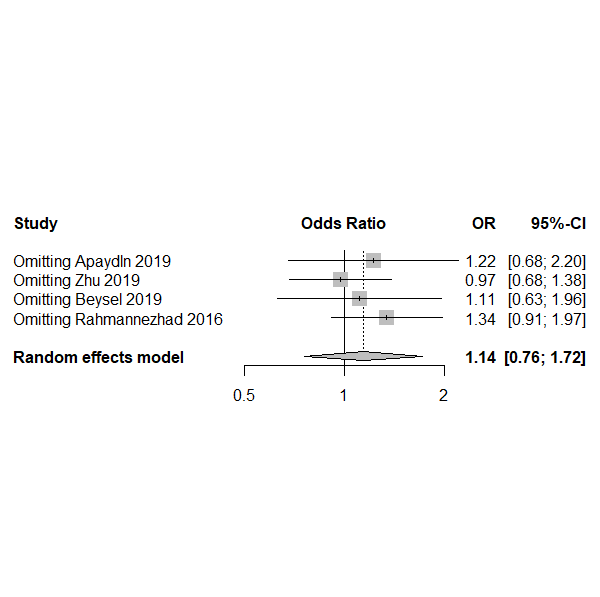


rs731236 co-dominant model (CT vs TT)


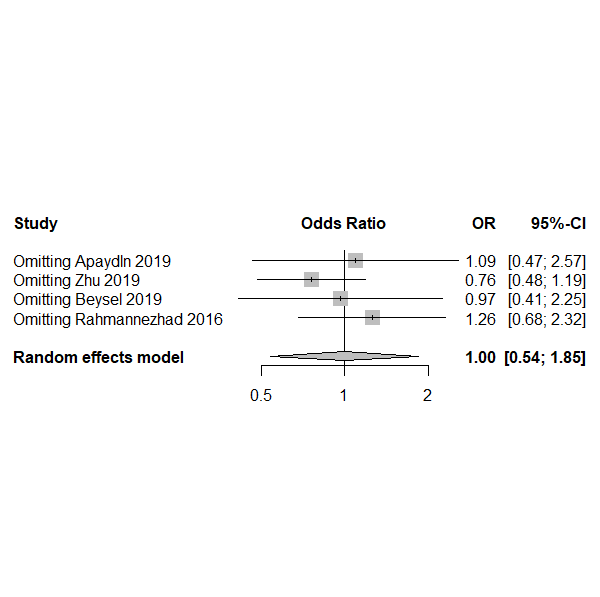


rs731236 dominant model (CT+ CC vs TT)


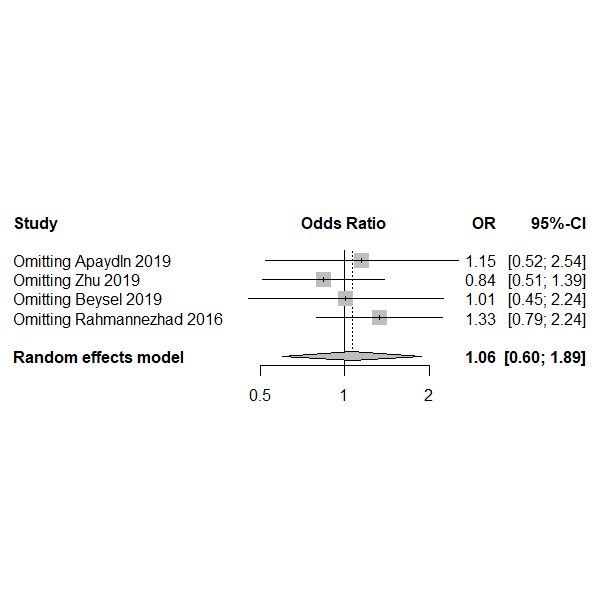

Supplement: Supplementary file 1 [file ijerph-18-00205-s001.zip › ijerph-1040959 - Supplementary/ijerph-1040959/Supplementary 2.docx]
